# Non-Empirical Large-Scale Search for Optical Metasurfaces

**DOI:** 10.3390/nano10091739

**Published:** 2020-09-02

**Authors:** Masanobu Iwanaga

**Affiliations:** Research Center for Functional Materials, National Institute for Materials Science (NIMS), 1-1 Namiki, Tsukuba 305-0044, Japan; iwanaga.masanobu@nims.go.jp

**Keywords:** non-empirical search, large-scale search, structural search, all-dielectric metasurface, spectral analyzer, metagrating, metadevice

## Abstract

Metasurfaces are artificially designed, on-top, thin structures on bulk substrates, realizing various functions in recent years. Most metasurfaces have been conceived of for attaining optical functions, based on elaborate human knowledge-based designs for complex structures. Here, we introduce a method for a non-empirical, large-scale structural search to find optical metasurfaces, which enable us to access intended functions without depending on human knowledge and experience. This method is different from the optimization and modification reported so far. To illustrate the outputs in the non-empirical search, we show unpredictable, optically high-performance, all-dielectric metasurfaces found in the machine search. As an extension of the finding of a higher order diffractive structure, we furthermore show a light-focusing metadevice, which is diffraction-limited and has the unique feature that the focal length is almost invariant even when the distance from the incident spot to the metadevice largely varies.

## 1. Introduction

Designs for metasurfaces have been mostly human knowledge based and optimizations from building blocks known previously. For example, metalenses [1,2,3,4,5,6], holograms [7,8,9], light absorbers [10,11,12,13,14,15,16], metagratings [17,18,19], luminescence-enhancing metasurfaces [20,21,22,23,24,25,26], and so on, were fabricated in such ways. The designs for metasurfaces with a particular function are generally elaborate and limited to the domain conceivable from human knowledge, though they are sometimes assisted by optimization algorithms. However, the unit cells or supercells in the metasurfaces have hundreds to hundreds-of-thousands of grids; therefore, even if each grid has only two possible options, e.g., air or a material, the total possible designs are $2^{100}$ to $2^{100,000}$, being huge numbers and far beyond human conceivable structures. Thus, there is much room to explore unknown functional metasurfaces.

In this article, we report a non-empirical search for optical metasurfaces. The empirical search is based on building blocks that are already regarded as being useful in human knowledge. Here, we define the non-empirical search as the search that starts without any building blocks. Instead of the building blocks, we provide a rule that automatically produces structures without preconception.

In the non-empirical search, we conducted large-scale numerical searches for two-dimensional (2D) and one-dimensional (1D) all-dielectric metasurfaces. In both the 2D and 1D cases, we found high-performance metasurfaces in targeted optical quantities. Functional metadevices making use of the metasurfaces found are newly designed. The procedure of the non-empirical large-scale search is described in Section 2, the results for the 2D metasurfaces in Section 3, those for the 1D metasurfaces in Section 4, a new metadevice based on the 1D result in Section 5, concluding remarks in Section 6, and additional information on the methods in Section 7.

## 2. Non-Empirical Search Procedure

**Figure 1:**
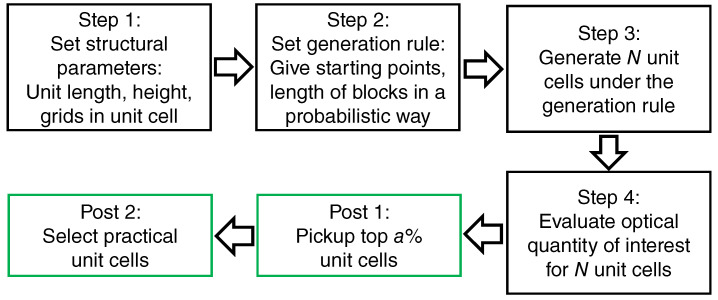
A sequence of procedures for the non-empirical structural search in this study. The structural search processes are indicated with black boxes, and post processes are indicated with green boxes.

Figure 1 shows the procedure of the non-empirical search, which starts by setting the structural parameters. We first assumed that the metasurfaces are single-layer 1D or 2D periodic structures on thick transparent substrates. Each step is as follows.

**Figure 2:**
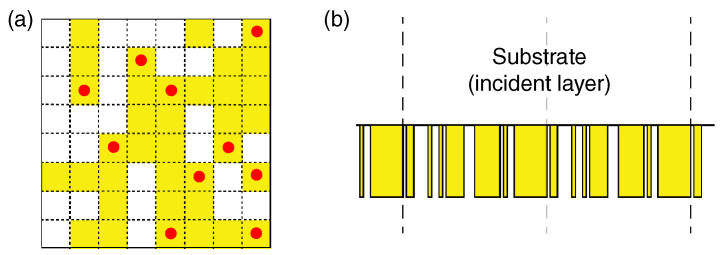
Automatically generated unit cells. (**a**) A 2D example; (**b**) a 1D example. The unit cell is located between the vertical broken lines.

Step 1 The dimensions and grids of unit cells and the height of the metasurfaces were initially set.
Step 2 Next, the generation rule for the unit cells was set. As one of the ways, rectangular blocks were generated by randomly choosing starting points such as the red dots in Figure 2a and by placing rectangular blocks in a probabilistic way. Concrete examples are described in Section 2.1.
Step 3 Generating *N* unit cells in an automatic way following the generation rule. We set *N* in a range from 500 to 1000 in most runs.
Step 4 We evaluated the optical quantity of interest for the *N* unit cells. Reflectance (R), transmittance, and diffraction efficiency were set as the optical quantities, which were precisely computed using the Rigorous Coupled-Wave Analysis (RCWA) method [27] combined with the scattering matrix algorithm [28]. The details of the numerical methods are described in Section 7.

In the post processes indicated by the green boxes in Figure 1, we pick up the top *a*% unit cells in terms of the optical quantity of interest (Post 1). Typically, we set a≤5. Among the pickup unit cells, practical unit cells are selected from the viewpoint of nanofabrication (Post 2); this last step is based on human knowledge and judgment, taking account of the practical ability in nanofabrication.

Large-scale search means that a large number of *N* is set. In this study, we set N ∼ 500 for the 2D unit cell search and N ∼ 1000 for the 1D unit cell search in accordance with available computational resources. In many implementations of the non-empirical search, good unit cells were mostly found to exhibit the high-performance targeted optical quantities. On the 1D structures, larger *N* than 1000 can be taken, but we found that the pickup unit cells are almost the same level as those found in the searches with N ∼ 1000.

The non-empirical search in this study is different from so-called optimization algorithms [29,30,31,32], which were frequently applied for photonic crystal waveguides. The optimization algorithms start from a known functional structure that has not yet been optimized and intend to optimize a particular performance. The optimization algorithms assume that there is already a good structure. In contrast, the non-empirical search does not assume any structure in advance and starts from zero, meaning that it does not allow us to have any idea about a good structure. We stress again that the non-empirical search can start without any building block that might be promising.

Furthermore, inverse designs tried to find functional waveguides [33] and plasmonic metasurfaces [34]. In these cases, transmission (or reflection) was set as a target quantity. In the designs, the target quantity was optimized using a mathematical algorithm [33] or a small number of given unit structures [34]. Although the examples using the inverse designs have not been many to date, the designs are likely to explore a small portion of the diverse possibilities.

The genetic algorithm could implement a non-empirical search when the initial set of genes (in this case, unit cells) is generated without relying on human knowledge-based structures. In some reports [35,36,37,38], electromagnetic nano-/micro-structures were explored using the genetic algorithm, which tends to require a longer implementation time than the present non-empirical search because the former generally needs many generations (or repetitions of the search) to yield good unit cells of many grids [39]. Due to the practical limitation of computational resources, the number of genes in each generation was limited to the small number of 600, even when unit cells consist of only 15 grids [38]. The non-empirical search in this study is able to explore a larger number of structures since it does not need repeated computations that take a long time. Details on the non-empirical structural search are described in the following subsections.

### 2.1. Automatically Generated Unit Cells and Constituent Materials

As a constituent material of the metasurfaces in structural search, a transparent dielectric with a refractive index of 2.3 was assumed. TiO${}_{2}$, GaN, Si${}_{3}$N${}_{4}$, and so on, almost satisfy the refractive-index condition in the visible range [40,41,42,43]. Such dielectrics are compatible with top-down nanolithography, being employed to realize dielectric metasurfaces of a high aspect ratio (height/width) of more then 10 [4,44]. The substrate for the metasurfaces was assumed to be SiO${}_{2}$, being transparent and having a refractive index of 1.46.

Figure 2a,b shows the schematics of unit cells in the 2D and 1D structures, respectively, which were generated in the non-empirical search. In the 2D structure, ten starting points (red dots in Figure 2a) were randomly chosen in the 8×8 grid unit cell. Note that the unit cell has $2^{64}\approx 2.62\times 10^{5}$ possible combinations in total. After setting the 10 starting points (red dots) in Figure 2a, rectangular blocks of 1×4 grids were placed, allowing the blocks to overlap; then, the filling ratio was approximately 0.6, which is a moderate value and tends to produce functional structures. The four orientations of the rectangular rods from the starting points were also determined randomly. In this procedure, the number of starting points and the shape of rectangular blocks were options when we implemented the structural search. A basic concept for the probabilistic generation of the unit cell is to avoid mere random generation because totally random generation tends to result in anti-reflection structures, which have been already conceived of using anti-reflection texture structures [45] and other nanostructures [46]. The rectangular shape of 1×4 grids played a role in avoiding randomly scattered, disconnected 1×1 grids.

Other generation rules are of course possible. For instance, the length of the rectangular blocks was set in a probabilistic way; the four types of 1×2, 1×3, 1×4, and 1×5 silicon blocks were chosen as 10%, 30%, 40%, and 20%, respectively, in a 20×20 grid unit cell of 800 nm squared. The filling ratio was approximate 0.7 on average. Large light absorption metasurfaces were found in the search, though the results are not shown here.

The way to generate 1D unit cells in Figure 2b was rather simpler than the 2D unit cells. The period length was set to 800 nm, indicated by the two nearest vertical dashed lines. The height was set to 400 nm. The unit cell was divided into 40 grids; then, approximately $1.11\times 10^{12}$ combinations are possible. The starting points were set to 20 and randomly set. Blocks of 1, 2, 3, and 4 grid lengths were generated at 10%, 40%, 40%, and 10%, respectively. Then, the filling ratio was approximately 0.7. The 1D metasurface (or metagrating) in Figure 2b is addressed later (Section 4).

### 2.2. Number of Test Unit Cells

To implement a large-scale search, the computational resources are a key factor. We implemented the structural search on a supercomputer, SX-ACE, in Tohoku University, which has multi-core, vector processors of 276 GFLOPS at the theoretical maximum and allows 1024 MPI runs at the maximum.

Hundreds of to a thousand unit cells (N=500 to 1000) were evaluated in the structural search. We implemented the structural search under 128 or 256 MPI runs, which enabled us to have an optical spectrum at one step and to grasp the basic optical properties of the auto-generated unit cells.

## 3. 2D All-Dielectric Metasurfaces

In this section, we address a set of results in a structural search for 2D all-dielectric metasurfaces. The unit cell was set to 560×560 nm${}^{2}$ with 8×8 grids, as shown in Figure 2a, and the height was 680 nm. The constituent material was set to have a refractive index of 2.3. The dimensions in the unit cells were often reported in actually fabricated metasurfaces [3,5,9,47].

### 3.1. Evaluation of Optical Performance

**Figure 3:**
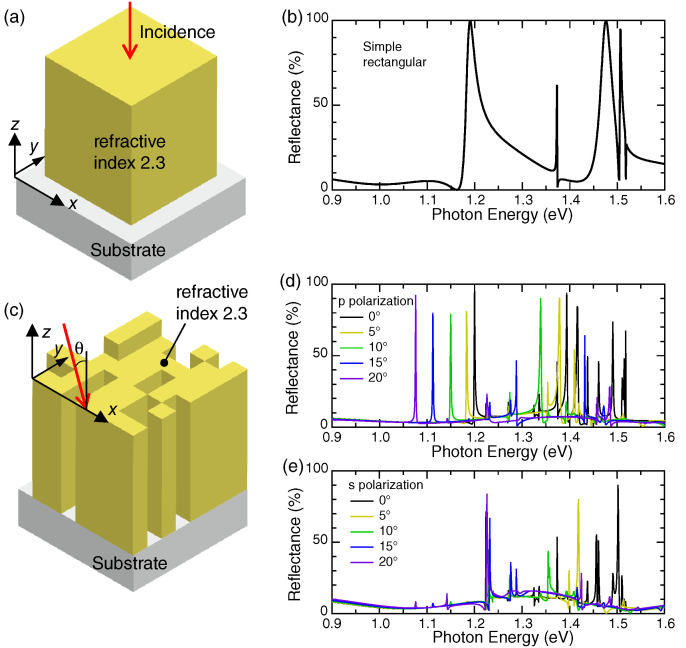
(**a**,**b**) A unit cell of a simple square-lattice metasurface of nano-rods and the R spectrum at the normal incidence, respectively; the periodic length is 560 nm; the side of the material with a refractive index of 2.3 (yellow) is 420 nm; and the height is 680 nm; (**c**–**e**) a high-performance 2D unit cell found in the non-empirical search; (**c**) the unit cell and incident configuration are depicted in a 3D manner; the period length is 560 nm, and the height of the unit cell is 680 mn; (**d**,**e**) linearly polarized R spectra at p and s polarizations are shown, respectively.

Figure 3a shows a simple metasurface of a square lattice of nano-rods, which has dimensions of 420 × 420 × 680 nm${}^{3}$ and whose height is 680 nm. The nano-rods comprise a transparent dielectric with a refractive index of 2.3. The metasurface is assumed to be on a transparent substrate with a refractive index of 1.46, such as SiO${}_{2}$.

The numerically calculated R spectrum of the metasurface at the normal incidence is shown in Figure 3b. Almost perfect reflection peaks appear at 1.190 and 1.476 eV with rather broad line widths. Especially, the perfect reflection peak at 1.190 eV is asymmetric for photon energy, indicating Fano resonance [20,48], coming from the interaction between a discrete Mie resonance in the rectangular dielectric nano-rods and photonic continuum.

A three-dimensional (3D) illustration of a metasurface found in the non-empirical search is shown in Figure 3c. The incident light configuration is drawn with a red arrow; we set the incidence in the xz plane. The R spectra of the metasurface under p and s polarizations are shown in Figure 3d,e, respectively; p polarization means that the incident electric-field vector $E_{\mathrm{in}}$ is in the xz plane and s polarization that $E_{\mathrm{in}}$ is parallel to the *y* axis. Incident angle θ was changed from $0^{{}^{\circ}}$ to $20^{{}^{\circ}}$ at $5^{{}^{\circ}}$ steps. Obviously, narrow R peaks appear in the range from 1.076 to 1.200 eV under p polarization, whereas any such prominent R peak does not appear under s polarization. Thus, it was found that the metasurface has a highly polarized spectral feature. We also note that the line width of the R peaks at p polarization is far narrower than that of the R peak at 1.190 eV in the simple metasurface in Figure 3a.

Other narrow R peaks appear in the range from 1.250 to 1.550 eV. The peaks are related to diffraction channels because the lowest diffraction channel is open at 1.516 eV (i.e., 817.6 nm in wavelength) under normal incidence. Since the modes are many in the range greater than 1.250 eV, the examination is too detailed and demanding. Let us focus our interest on the resonant mode at 1.076–1.200 eV, examine the origin, and then construct a compact spectral analyzer employing the found metasurface in the following.

**Figure 4:**
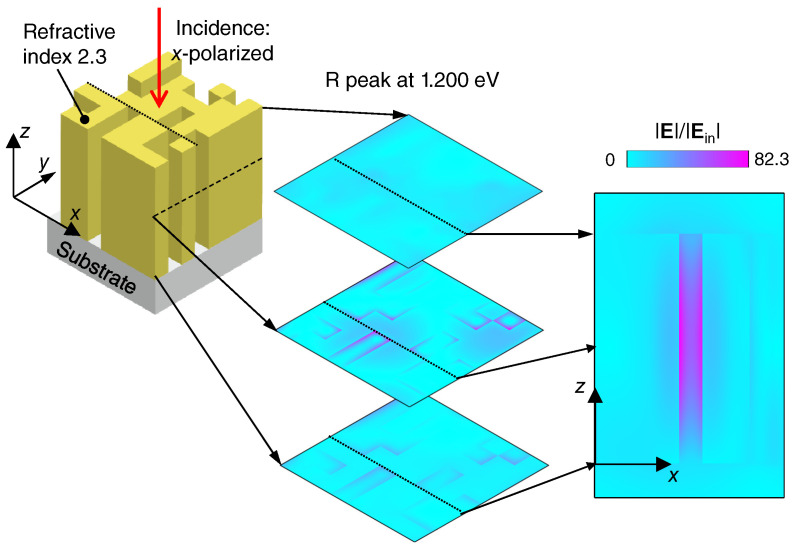
Resonant electric-field distribution in the 2D metasurface found in the non-empirical search. The absolute value |E| is plotted, which is normalized by incident $|E_{\mathrm{in}}|$. Incidence at 1.200 eV was set to be normal to the xy plane and *x*-polarized. Three xy sections are shown in the middle, and an xz section is at the right. The *z* positions at the half height are indicated by arrows, and broken lines indicate the position of the section. One xz section is shown at the right, and the section is indicated by dotted lines at the left and middle in the xy planes. The scale bar is in common with the middle and right panels.

The resonant feature is visualized in Figure 4, presenting the electric field. We show the absolute value of electric field |E| at 1.200 eV and the *x*-polarized normal incidence. The configuration is shown in Figure 4 (left). Three xy sections of |E| are shown at the center; the *z* positions, which are the top, middle, and bottom sections of the unit cell, are indicated by arrows. A xz section is shown at the right; the section is taken at the dotted-line position in the left and center panels. The most resonant enhanced |E| distribution is seen in the xz section. The scale bar is in common with the center and right panels. Note that the resonant electric field is significantly enhanced, and the ratio of $\left|E|/\right|E_{\mathrm{in}}|$ reaches 82.3 at the maximum where $|E_{\mathrm{in}}|$ denotes the absolute value of incident light. Considering the ratio of electric field intensity ${\left|E\right|}^{2}$, it reaches 6773 at the maximum, which exhibits significant resonant enhancement. The most prominent enhancement takes place in the gap between the dielectric block, as shown in Figure 4. The enhanced electric field does not appear at any dielectric sharp corner, which is a good signature for actual nanofabrication since a sharp corner is usually difficult to realize even when we exploit the best contemporary top-down technique.

**Figure 5:**
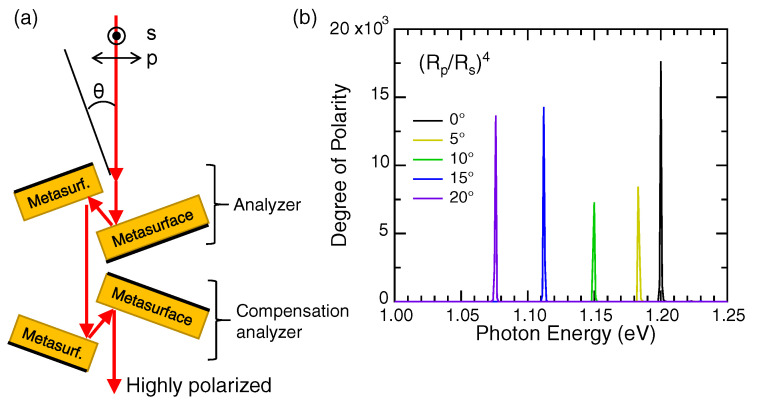
(**a**) Schematic of a compact, high-throughput, on-axis spectroscopic analyzer, composed of the pairs of metasurfaces in Figure 3c; (**b**) degree of polarity by the spectroscopic analyzer in (**a**), which is equal to a ratio of ${\left(R_{p}/R_{s}\right)}^{4}$. The incident angle θ in (**a**) varies from $0^{{}^{\circ}}$ to $20^{{}^{\circ}}$ by rotating the analyzer by θ and the compensation analyzer by −θ. The FWHMs of prominent peaks are less than 1 nm.

Figure 5a illustrates a compact, high-throughput, on-axis spectroscopic analyzer, which is constructed using the 2D metasurface in Figure 4. Four pieces of the 2D metasurface form the spectroscopic analyzer such that the pair of two pieces works as an analyzer. A pair is placed to compensate beam shift due to the first analyzer, so that the transmitted light through the spectroscopic analyzer is always on-axis to the incident beam and highly polarized. The degree of polarity by the spectroscopic analyzer is shown in Figure 5b. The degree of polarity is equivalent to the four-times reflection ratio of ${\left(R_{p}/R_{s}\right)}^{4}$, where $R_{p}$ and $R_{s}$ denote p- and s-polarized reflectance, respectively. The incident polarization is indicated in Figure 5a. We note that the degree of polarity exceeds 5000 for incident angles from 0${}^{{}^{\circ}}$ to 20${}^{{}^{\circ}}$ and that the highly polarized transmission light becomes monochromatic with a narrow bandwidth less than 1 nm in the Full-Width Half Maximum (FWHM). Thus, it is justified to call the device a spectroscopic analyzer. The throughput for the p-polarized incidence is greater then 40% in the whole energy range from 1.07 to 1.20 eV. The throughput meets the application to produce monochromatic, polarized light from light-emitting diodes or broadband lasers.

In a practical configuration, the first analyzer and the compensation analyzer are place on the rotation device, keeping the on-axis operation of the metadevice, that is when rotating the first analyzer by θ, the compensation analyzer is rotated by −θ. Then, rapid scanning of the spectroscopic analyzer can be realized. We note that the back sides of the metasurface substrates are assumed to be blacker (or more light absorptive) to avoid unnecessary light scattering.

## 4. 1D Transmissive Metasurfaces

1D metasurfaces are addressed in this section. When diffraction is a targeted quantity of interest, they are often called metagratings. The first-order diffraction efficiency was optimized, and metagratings have been frequently reported so far [17,18,19]. Here, we apply the non-empirical search for 1D metasurfaces of large higher order diffraction efficiency; concretely, the metagrating found of third-order diffraction efficiency more than 80% is specified.

**Figure 6:**
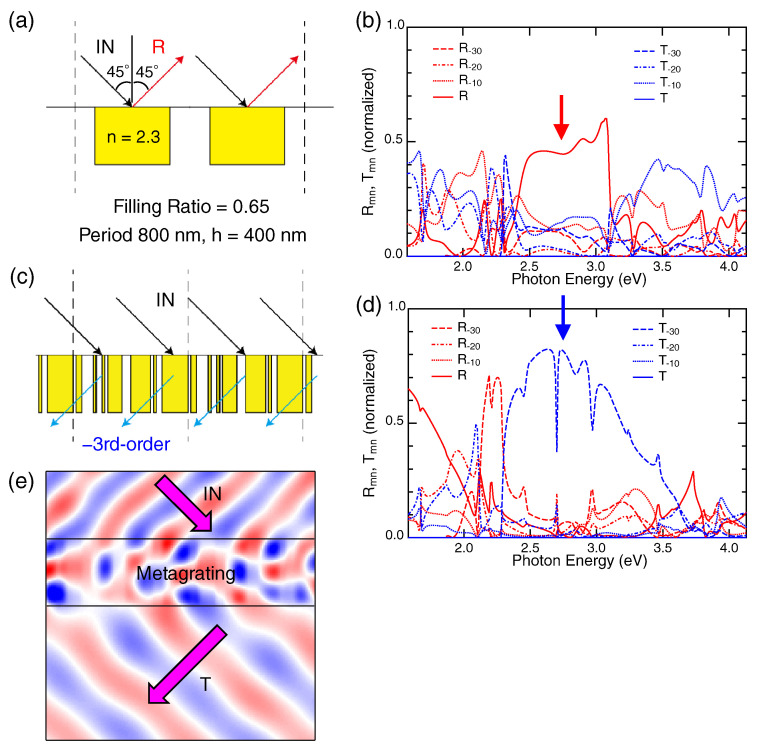
(**a**) A lamella grating of a material with a refractive index of 2.3. The periodic length is 800 nm, the height 400 nm, and the filling ratio in the unit cell 0.65. (**b**) Diffraction components of the lamella grating in (**a**). The incident angle was 45${}^{{}^{\circ}}$. All the open channels are shown; the highest order is the third-order T${}_{-30}$ and R${}_{-30}$. R denotes the zeroth-order reflection, that is ordinary reflection. R${}_{m0}$ and T${}_{m0}$ are represented with red and blue curves, respectively. Dotted curves shows the m=1 component, dot-and-broken curves m=2, and dashed curves m=3. (**c**) A high-performance 1D metasurface (metagrating) found in the non-empirical search. The nearest pair of vertical dashed lines indicates the unit cell. (**d**) Diffraction components of the metagrating in (**c**). All the open channels are shown at an incident angle of 45${}^{{}^{\circ}}$. The third-order transmission $T_{-30}$ is dominant at 2.5–3.0 eV. The way of curve presentation is in common with that in (**b**). (**e**) A snapshot of the magnetic-field distribution in the metagrating (**c**). The incident light is 2.75 eV, indicated by the blue arrow in (**d**). Effective wave vectors in the incident and transmission layers are drawn with magenta arrows, to guide the eye.

Before describing the metagrating, a typical lamella grating in Figure 6a is addressed to grasp the usual diffraction responses by gratings. The period length and height were set to 800 and 400 nm, respectively. The filling ratio of a dielectric with a refractive index of n=2.3 in the unit cell was set to 0.65, meaning that the width of the dielectric rod is 65% of the periodic length, i.e., 520 nm. An incident layer was assumed to be a transparent material with a refractive index of 1.46 such as SiO${}_{2}$, and the transmission layer was air with a refractive index of 1.00027. The plane of incidence was set to the plane perpendicular to the dielectric rods.

The diffraction efficiencies of the lamella grating at incident angle set to 45${}^{{}^{\circ}}$ are shown in Figure 6b, where all the open diffraction channels up to the third order are shown. Red curves denote reflective components R${}_{m0}$ and blue curves transmissive components T${}_{m0}$ (m=1,2,3). We note that the zeroth-order components are ordinary reflectance and transmittance, being simply written as R and T, respectively. The largest component is R in the range from 2.5 to 3.0 eV (red solid curve). Note that the incident condition is the total reflection configuration that forbids non-zero T. Among the diffraction components (m≥1), the first-order ones take the largest values in the same range. In addition, the higher order components (m≥2) appear together at an amount comparable to the first-order; therefore, the diffraction responses are rather complicated and unsuitable to use the grating for spectroscopic purposes.

Figure 6c shows a unit cell of the metagrating found in the non-empirical search, described in Section 2. We picked up the unit cell from the result through the post process in Figure 1. As an optical quantity, the third-order component T${}_{-30}$ was examined. The unit cell in Figure 6c was the best one in the search whose details are noted in Section 2.1. The T${}_{-30}$ at 2.75 eV of the top five unit cells are listed in Table 1, together with other components; the values of the components are normalized, taking values between zero and one. We mention that the unit cells exhibiting $T_{-30}>0.7$ were limited to the top 19 ones, which were 3.8% among the all unit cells explored in the search. Thus, it turns out that the non-empirical search worked quite efficiently, in spite of the very small explored portion $N/2^{40}\approx 9.00\times 10^{-10}$.

The diffraction spectra of the unit cell in Figure 6c are plotted in Figure 6d. The incident angle was 45${}^{{}^{\circ}}$, and the incidence was p-polarized. It is evident that the T${}_{-30}$ component is dominant in the range from 2.5 to 3.0 eV. In contrast to the spectra of the lamella grating (Figure 6b), the zeroth-order reflectance R was suppressed. To visualize the T${}_{-30}$-dominant effect at 2.75 eV and the incident angle of 45${}^{{}^{\circ}}$ (blue arrow in Figure 6d), we evaluated the magnetic field distribution, which is a good quantity for p (or Transverse Magnetic (TM)) polarization. Figure 6e shows a snapshot of the distribution; as a result, a major part of the electromagnetic wave propagation exhibits an effectively negative refraction, which is indicated by effective wave vectors (magenta arrows). The wave manipulation by the metagrating is quite unique.

## 5. Focal Length-Invariant Metadevice

**Figure 7:**
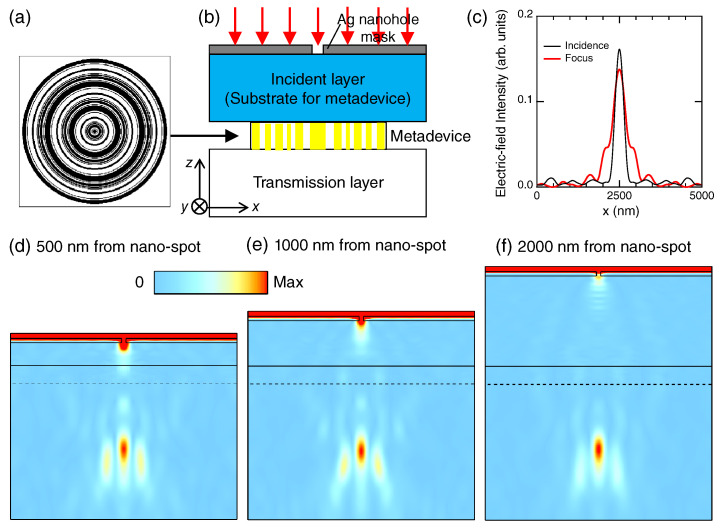
Focusing length-invariant metadevice made of the 1D metasurface found in Section 4. (**a**) The focusing metadevice structure; (**b**) a test configuration for the metadevice, in which the Ag nanohole mask is introduced to induce an incident light spot; red arrows denote incident light; (**c**) electric field intensity profiles at incident and focal spots; the black curve shows the section of the incident spot and red that of the focal spot; (**d**–**f**) the metadevice is placed at a distance of 500, 1000, and 2000 nm from the 100 nm width nanospot, respectively; Poynting fluxes |S| are plotted in the configuration (**b**); in (**c**–**f**), the incident photon energy was set to be 2.75 eV.

In this section, we construct a focusing metadevice made of the 1D metasurfaces, which exhibit effectively a negative refraction, as shown in Section 4. Figure 7a shows the metadevice structure of a circular shape. From the center along the radius, the 1D metasurface structure is placed. The side of the square box is 5 μm.

Figure 7b depicts a test configuration for the focusing effect. A light spot was produced using a Ag nanohole mask of a 100 nm diameter circular hole in 5×5μm${}^{2}$. We set the mask and metadevice to be placed periodically at 5 μm along the *x* and *y* axes, in order to employ the RCWA method. The incident layer to the metadevice is the substrate of the metadevice, being assumed to be SiO${}_{2}$ with a refractive index of 1.46. The transmission layer is air with a refractive index of 1.00027.

Figure 7c shows section profiles of electric-field intensities in incident and transmission layers in the case of a 2000 nm incident layer (Figure 7f). Incident photon energy was set to 2.75 eV. Black and red curves show the section profiles at the incident and focusing spots, respectively; the profile in the incident layer was taken at the position 100 nm away from the Ag nanohole end (i.e., the interface of the Ag mask with the incident layer), and the profile in the transmission layer was at the spot distant by 1480 nm from the metadevice end (i.e., the interface of the metadevice with the transmission layer), which is represented with the dashed line in Figure 7f. The FWHM of the incident spot is 306 nm, and the FWHM of the focusing spot is 460 nm. This result strongly suggests that the metadevice has a quite good focusing capability under the diffraction-limited condition because an FWHM relation including refractive indices holds such that $306\times n_{{\mathrm{SiO}}_{2}}\approx 460\times n_{\mathrm{air}}$ where $n_{{\mathrm{SiO}}_{2}}=1.46$ and $n_{\mathrm{air}}=1.00027$. Furthermore, we estimated the throughput of the metadevice by taking the peak ratio of the electric field intensities in Figure 7c and found that 85.3% intensity is focused by the metadevice; therefore, we can declare that the metadevice is quite high-throughput.

Focusing effects by the metadevice are shown for three cases of 500, 1000, and 2000 nm incident layers in Figure 7d–f, respectively. We define the incident layer as a layer between the Ag nanohole mask and the metadevice. Poynting fluxes |S| are plotted at an incidence of 2.75 eV. We set the incidence |S|=1 (red arrows). It is evidently seen that the incident light spot induced by the Ag nanohole forms a focusing spot again in the transmission layer in each case. At the focusing spots, the maxima of |S| are 0.056, 0.067, and 0.153 in Figure 7d–f, respectively. Note that, since the incidence is substantially reduced by the Ag nanohole, it is reasonable that the Poynting fluxes become substantially smaller than one. As the thickness of the incident layer to the metadevice gets larger, the Poynting flux at the focusing spot becomes larger; this tendency probably comes from the light through the Ag nanohole having larger wavenumber components as the thickness is larger, so that the focusing effect becomes more prominent.

We point out in particular that the way of focusing light is different from conventional refraction lenses; that is, the focusing spot is located at almost the same distance from the metadevice end (dashed lines), irrespective of the distance from the light source spot to the metadevice. The distance from the metadevice end to the focusing spot is 1450, 1480, and 1430 nm in Figure 7d–f, respectively, being almost the same in the three cases. Such short-distance focusing is hardly realized by conventional refraction lenses. In addition, as is well known, the conventional lenses change the focal length in accordance with the distance from the incident spot to the lenses, so that it is always necessary to adjust the focal length to detect the light. In contrast, the metadevice shown here is free from the adjustment, enabling us to easily detect the light owing to the focal length (or working distance) invariance.

Thus, the metadevice exhibits a unique and convenient feature, which has not been reported so far to our knowledge. We note that, in order to discriminate this metadevice from the conventional refraction lens, the metadevice is suitable not to be simply called a lens (or metalens), but a focusing device with working distance invariance. This is probably realized owing to mechanism by which the metadevice selectively and effectively refracts the incoming light using the T${}_{-30}$ channel; therefore, for incident photon energy in 2.5–3.0 eV, the focal spot always appears in a similar manner. In fact, we confirmed that the focusing effect is also obtained at 2.50 eV as well as the case of 2.75 eV (Figure 7).

## 6. Concluding Remarks

We implemented a non-empirical, large-scale structural search for all-dielectric optical metasurfaces and addressed a few high-performance metasurfaces that were picked up among many good metasurfaces found in the search. The picked up metasurfaces were made use of to construct novel optical devices such as the compact, on-axis wavelength analyzer and the diffraction-limited, light-focusing metadevice with the unique feature of focusing-length invariance. The present strategy to create new optical devices started from the non-empirical search and enabled building the optical devices made of the high-performance metasurfaces found in the search. Although the non-empirical search was quite productive, we note that the explored part remains extremely small with respected to all the possibilities, which means that there is much room to find metasurfaces of other optical functions.

Symbolically, the present non-empirical search starts at zero (no knowledge in advance) and yields one (high-performance metasurfaces). Additionally, the optimization techniques referred to in Section 2 could be used to gain incremental improvements, as they have done so far.

## 7. Methods

We here describe the numerical details of the RCWA method [27] and the scattering matrix algorithm [28]. When Maxwell equations are resolved for periodic objects, it is a suitable way to solve the Fourier-represented Maxwell equations, which are linear equations comprising the Fourier coefficients of electromagnetic components and are mathematically equivalent to the original Maxwell equations. By truncating the Fourier expansion, the Fourier-represented Maxwell equations become finite equations, which can be handled numerically. The RCWA method was conceived of to make stable implementations possible for arbitrary 2D layers with periodicity.

3D objects are always divided into stacked 2D layers. To combine the electromagnetic modes in the 2D layers, the scattering matrix algorithm is useful because it avoids exponentially growing components along the directions perpendicular to the 2D layers and ensures stable numerical implementations.

The material parameters, especially complex permittivities, were taken from representative values for air, SiO${}_{2}$, TiO${}_{2}$, Si${}_{3}$N${}_{4}$, and GaN [40]. Further extended details in this section and various numerical examples are provided in [20].

## Figures and Tables

**Table 1:** Top 5 values of T${}_{-30}$ at 2.75 eV in the non-empirical search for the metagrating in Figure 6c. The mark * denotes that data are not available since they were of less interest and not collected.

Rank	T${}_{-30}$	T${}_{-20}$	T${}_{-10}$	R
1	0.8146	0.0135	0.0148	0.0125
2	0.7928	0.0132	0.0294	0.0304
3	0.7529	*	*	*
4	0.7414	*	*	*
5	0.7363	*	*	*

## Abbreviations

The following abbreviations are used in this article:

1D	One-Dimensional
2D	Two-Dimensional
3D	Three-Dimensional
FWHM	Full-Width Half Maximum
MPI	Multi-Parallel Implementation
R	Reflectance
RCWA	Rigorous Coupled-Wave Analysis
